# Myanmar's coup risks a flood of vaccine-preventable disease

**DOI:** 10.7189/jogh.12.03060

**Published:** 2022-09-03

**Authors:** Kaung Suu Lwin, Cyrus Ghaznavi, Khine Lae Win, Stuart Gilmour, Masahiro Hashizume, Shuhei Nomura

**Affiliations:** 1 Department of Global Health Policy, Graduate School of Medicine, The University of Tokyo, Tokyo, Japan; 2 Department of Health Policy and Management, School of Medicine, Keio University, Tokyo, Japan; 3 Medical Education Program, Washington University School of Medicine in Saint Louis, Missouri, USA; 4 Department of Mental Health, Graduate School of Public Health, The University of Tokyo, Tokyo, Japan; 5 Graduate School of Public Health, St. Luke's International University, Chuo-ku, Tokyo, Japan; 6 Tokyo Foundation for Policy Research, Tokyo, Japan

In line with the Sustainable Development Goals (SDGs), the reduction of the under-5 mortality rate to ≤25 deaths per 1000 live births by 2030 is the primary target of Myanmar’s national strategic plan for newborn and child health [1]. Myanmar made considerable strides in decreasing under-5 mortality with a 61% reduction between 1990 and 2019; however, the rate remained high at 45 per 1000 live births as of 2019 and more progress is needed [2]. In Myanmar, more than 45 000 under-5 children died in 2017 primarily due to newborn causes (46.7%), pneumonia (15.1%), diarrhea (8.7%) and injuries (8.5%) [2]. Because immunization is one of the most cost-effective and efficient ways to improve child health [3], Myanmar’s 1978 Expanded Program on Immunization (EPI) [4] was later broadened between 2016 and 2020 to include pneumococcal conjugate vaccines (PCVs), Japanese encephalitis (JE) and rotavirus vaccines [5]. Since then, EPI has been supplying nine types of vaccines for children in Myanmar (**Table 1**) [4,5].

**Table 1:** Childhood immunization schedule in Myanmar, 2020*

Vaccine	Age of administration
BCG	Birth to 2 mo
HepB	Birth to 24 h
DTP-Hib-HepB (pentavalent)	2 mo, 4 mo, and 6 mo
OPV	2 mo, 4 mo, and 6 mo
IPV	4 mo
PCV	2 mo, 4 mo, and 6 mo
MR	9 mo and 18 mo
JE_LiveAtd	9 mo
Rotavirus	2 mo and 4 mo

BCG – Bacille Calmette-Guérin, HepB – hepatitis B vaccine, DTP – diphtheria-tetanus-pertussis, Hib – *Haemophilus influenza* type b, OPV – oral polio vaccine, IPV – inactivated polio vaccine, PCV – pneumococcal vaccine, MR –combined measles and rubella vaccine, JE_LiveAtd – live-attenuated Japanese encephalitis vaccine, HPV – human papillomavirus, mo – months

*Source: WHO/UNICEF JRF, 2020.

**Figure 1:**
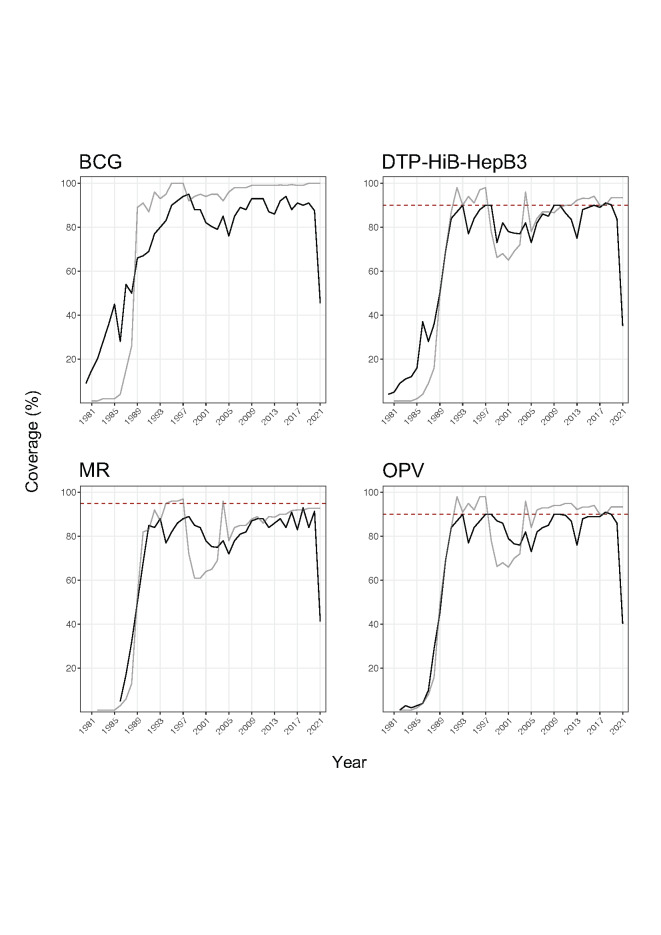
Time trends of childhood immunization coverage in Myanmar and Bangladesh, 1978-2021. BCG – Bacille Calmette-Guérin, DTP-Hib-HepB3 – three doses of pentavalent vaccine for diphtheria, tetanus-pertussis, Haemophilus influenza type b, and Hepatitis B, MR – the combined measles and rubella vaccine, OPV – oral polio vaccine. The dashed, red line shows the national, 2021 vaccination coverage target for Myanmar. The solid black and gray lines show vaccination coverage for Myanmar and Bangladesh, respectively. Data source: www.who.int.

The launch of EPI was followed by dramatic improvements in vaccination coverage in Myanmar (**Figure 1****)**. Even during the COVID-19 pandemic in 2020, vaccination coverage for tuberculosis, diphtheria, tetanus, pertussis, hepatitis B, measles, rubella, and polio was greater than 80%; the target coverage rate of 88% for 3 doses of the pentavalent vaccine (diphtheria, tetanus, pertussis, *Haemophilus influenza* type b, and hepatitis B) and oral polio vaccine (OPV) was reached successfully (**Figure 1**) [5]. Vaccine coverage for most childhood vaccine types in Myanmar was tracked with approximately corresponding rates to neighboring Bangladesh until the coup d’état happened in 2021 (**Figure 1**) [5]. EPI was suspended in many areas across Myanmar due to the subsequent unrest after the coup and the military’s brutal post-coup crackdown on civilians, including health care workers and health infrastructure [6]. As of January 2022, there have been at least 415 reported attacks and threats against health care workers and health infrastructure since the coup d’état took place in February 2021 [6]. Civilian access to clinical and preventive services has been impeded, and immunization programs are nearly non-functional [6]. As a result, EPI coverage dropped by 50% or more for many vaccination types in 2021 **(****Figure 1**), reversing several years’ worth of work advancing vaccine administration; vaccine coverage in 2021 reached some of the lowest rates seen in the past two decades [5].

As of May 2022, almost 700 000 civilians, including women and children, have been internally displaced (IDP) by crises related to the military coup [7]. UNICEF estimates that five million children in Myanmar need humanitarian assistance [7]. Many internally displaced children are living in overcrowded conditions in IDP camps where they cannot access essential health services, including immunization [7]. These children are at risk of malnutrition and may lack access to clean water and sanitation facilities [7]. Furthermore, the outbreak surveillance system collapsed amid the conflict [6,7]. Poor camp conditions, in conjunction with disruptions in the provision of and access to health care, have further highlighted the need for robust immunization programs. Failure to take immediate action may leave a generation of children at risk of serious health outcomes even if the political turmoil were to pass.

Overall declines in vaccine coverage in Myanmar not only impart health risks to children who cannot access immunizations. but also threaten the health of their communities. A key component of preventing disease outbreaks is the achievement of herd immunity [8]. Despite Myanmar’s declaration of polio-free status in 1996 [9], the risk of an outbreak of vaccine-derived poliovirus (VDPV) remains a concern when polio vaccine coverage becomes low. Since all types of childhood vaccine coverage became extremely low in 2021 [5], we are seriously concerned about the potential for outbreaks of vaccine-preventable diseases (VPDs), as was seen during the Syrian civil war with the emergence of polio outbreaks that even crossed borders into Iraq [10]. Myanmar is similarly at risk today, but the implications of VPDs resurging in IDP camps are not limited to Myanmar alone: cross-border spill over constitutes a significant global health concern.

The administrative responsibility of EPI is shared by multiple stakeholders, including the Ministry of Health, UNICEF, WHO, Gavi, the Vaccine Alliance and the implementing partners [4]. Han et al. have already warned the global health community that the military coup threatens the health and human security of Myanmar and its neighbors [11]. Myanmar was devasted by a catastrophic third wave of COVID-19 from July to September 2021. Due to the collapse of the public health care system and obstruction of health care by the Myanmar military, thousands of people died without getting access to needed health care [12-14]. There is clear evidence that children are suffering from the consequences of the collapse of Myanmar’s fragile health system.

**Figure:**
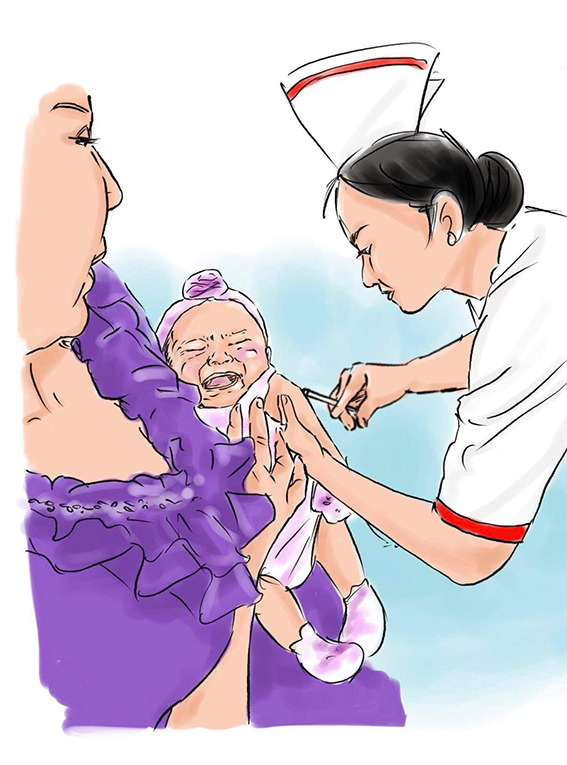
Photo: An illustration showing a child’s reaction to receiving vaccination. Source: Marn Kow (Yangon, Myanmar). Used with permission.

Urgent and sustainable action is required to prevent children from dying from VPDs before outbreaks are too advanced to control and spill over into surrounding nations. First, we reiterate our call to the Myanmar military to 1) immediately stop all forms of violence against civilians, including against health care workers and health infrastructure, 2) allow health services to resume throughout the country, including in IDP camps, 3) ensure safe and unimpeded access for international and local aid organizations to provide health and humanitarian assistance to IDPs, and 4) cooperate with multiple stakeholders to provide equitable access to health services (including childhood immunization) for all people in Myanmar, especially those in IDP camps who can only be reached by ethnic and community-based organizations or non-governmental and UN entities. We firmly call on UN organizations and the global community to ensure the full implementation of UN Security Council Resolution 2286, which strongly condemns attacks on health care personnel in conflict situations [15], and to fully adopt necessary measures to enhance the protection of and access to health care in Myanmar. We hope that UN and international organizations will negotiate with relevant parties to establish safe corridors to deliver essential health interventions, such as childhood immunization, ensure a continuous supply of vaccines and other commodities to sustainably provide childhood immunization and other essential health services, and support integrated outreach strategies ensuring that essential health services reach those in need. If the global community fails to act in a timely and compassionate manner, we may be reminded yet again that communicable diseases do not respect borders, and local health is ultimately global health.
